# Circulating MicroRNAs in Relation to *EGFR* Status and Survival of Lung Adenocarcinoma in Female Non-Smokers

**DOI:** 10.1371/journal.pone.0081408

**Published:** 2013-11-25

**Authors:** Huan Zhang, Yuliang Su, Fangxiu Xu, Jinyu Kong, Herbert Yu, Biyun Qian

**Affiliations:** 1 Department of Epidemiology and Biostatistics, Tianjin Medical University Cancer Institute and Hospital, National Clinical Research Center of Cancer, Tianjin, P.R. China; 2 Key Laboratory of Cancer Prevention and Therapy, Tianjin Medical University, Ministry of Education, Tianjin, P.R. China; 3 Epidemiology Program, University of Hawaii Cancer Center, Honolulu, Hawaii, United States of America; Roswell Park Cancer Institute, United States of America

## Abstract

**Objectives:**

Lung adenocarcinoma is considered a unique disease for Asian female non-smokers. We investigated whether plasma microRNA (miRNA) expression profiles are different by the EGFR status and are associated with survival outcomes of the patients.

**Methods:**

Using real-time RT-PCR, we analyzed the expression of 20 miRNAs in the plasma of 105 female patients with lung adenocarcinoma. Kaplan-Meier survival analysis and Cox proportional hazards regression were performed to determine the association between miRNA expression and overall survival. Time dependent receiver operating characteristic (ROC) analysis was also performed.

**Results:**

In the 20 miRNAs, *miR-122* were found differently expressed between wild and mutant *EGFR* carriers (*P*=0.018). Advanced disease stage and tumor metastasis were independently associated with poor prognosis of patients with lung adenocarcinoma (*P*=0.010 and 1.0×10^-4^). Plasma levels of *miR-195* and *miR-122* expression were also associated with overall survival in the patients, especially in those with advanced stage (HR=0.23, 95%CI:0.07-0.84; and HR=0.22, 95%CI:0.06-0.77) and *EGFR* mutation (HR=0.27, 95%CI:0.08-0.96; and HR=0.23, 95%CI=0.06-0.81). Moreover, a model including *miR-195, miR-122* may predict survival outcomes of female patients with lung adenocarcinoma (AUC=0.707).

**Conclusions:**

Circulating *miR-195* and *miR-122* may have prognostic values in predicting the overall survival as well as predicting *EGFR* mutation status in non-smoking female patients with lung adenocarcinoma. Measuring plasma levels of *miR-195* and *miR-122* may especially be useful in *EGFR* mutant patients with lung adenocarcinoma.

## Introduction

Lung cancer is one of the most lethal malignancies and the leading cause of cancer death in the world. The prevalence and mortality of the cancer is still rising. Lung cancer incidence in Asia varies from 14.3/100,000 in south central Asia to 50.4/100,000 in Japan and 61.4/100,000 in China[1]. Of the lung cancer cases diagnosed, approximately 85% are non-small cell lung cancer (NSCLC), which has poor prognosis and is difficult to treat. Despite years of research, the survival of NSCLC remains dismal, and the 5-year survival is only around 10% [2].

Although tobacco use is a well-established risk factor for lung cancer, the disease still occurs to patients who have no history of smoking. Recent evidence suggests that lung cancer incidence is rising among non-smokers, while the smoking population is declining in the developed countries [3,4]. Data from Asia also demonstrate that non-smokers constitute a significant proportion of NSCLC patients, who are quite different from smokers with NSCLC both in clinical and pathological characteristics. Non-smoking lung cancer has been considered a unique disease entity different from smoking one. Recently, it has been shown that patients who are responsive to the treatment of epidermal growth factor receptor tyrosine kinase inhibitors (EGFR-TKIs) are largely limited to Asian women who are non-smokers with adenocarcinomas. Patient cohort studies have investigated the differences in overall survival of lung cancer between smokers and non-smokers, and the results, however, were inconclusive due to possible confounding effects[5,6].

MicroRNAs are small non-coding RNAs with 18–25 nucleotides in length that regulate the activities of a large number of messenger RNAs and exert a wide range of biological functions, including behaving either as oncogenes or tumor suppressor genes. Accumulating evidence indicates that dysregulation of specific microRNAs contributes to a variety of diseases, most notably the development and progression of cancer, including lung cancer [7–9]. Recently, the potential for using circulating miRNAs as tumor biomarkers has been evaluated [10], but the results remain to be validated. In this study, we measured the expression of several microRNAs in the plasma of 105 non-smoking female lung adenocarcinoma patients and examined their associations with *EGFR* mutation and overall survival.

## Materials and Methods

### Patient enrollment

All patients in the study were recruited form Tianjin Medical University Cancer Hospital between January 2007 and June 2009. The patients were newly diagnosed and histologically confirmed primary non-small cell lung cancer. Patients with a previous medical history of cancer, radiotherapy or chemotherapy before surgery were excluded. The study was approved by an ethical review committee at Tianjin Medical University Cancer Hospital. After signing an informed consent form, all study participants were asked to complete a structured questionnaire with the help of trained research staff. The questionnaire collected information on demographic features and risk factors, including a family history of cancer and tobacco use (age at first use, years of smoking, cigarettes smoked per day, and age at quitting smoking if applicable). Clinical information such as histological type, tumor size, lymph node (LN) metastasis, disease stage, and post-surgical treatment including targeted therapy was extracted from medical records. 

During the study recruitment, we enrolled total 478 patients in the study. For the current investigation, only female patients who were diagnosed with adenocarcinoma and never smoked cigarettes were included. The patients who lost to follow-up in the first year were excluded. All patients were followed after surgery through clinical visit and regular telephone contact. Survival time was calculated from date of diagnosis to the date of death or last follow-up in July, 2012.

### Tissue and plasma samples

A blood sample (10 ml) was collected from each patient using an ethylene diamine tetraacetic acid (EDTA) vacutainer tube. Plasma was separated after centrifugation at 2,000 rpm for 20 min and then put into a liquid nitrogen tank for long-term storage until miRNA extraction and quantitative reverse transcription PCR (qRT-PCR). All matched tissue samples were histologically confirmed. These tissue samples were stored at -80°C.

### EGFR exon 18-21 mutation sequencing

DNA for *EGFR* sequencing test was extracted from the surgical specimen using the pheno-chloroform method. PCR was performed on 100 ng DNA samples to identify mutations in the *EGFR* exons 18-21, and Table S1 shown the *EGFR* primers. Then, PCR products were purified from agarose gel after electrophoresis (1% wt/vol), using the Ezgene^TM^ Gel/PCR Extraction Kit (Biomiga, San Diego, USA) according to the manufacture’s manual. Exons 18-21 of purified products were amplified in duplicate per exon and all variants were confirmed in both forward and reverse directions using the Big Dye Terminator Cycle Sequencing Kit (version 3.1, Applied Biosystems, Foster City, CA). The sequencing reaction was carried out in the ABI-3100 DNA sequencer (Applied Biosystems, Foster City, CA). All sequencing results were analyzed with the Sequencing Analysis software (SDS) version 5.4 (Applied Biosystems, Foster City, CA). Then, the sequences were compared with the standard sequences provided in the national center for biotechnology information (NCBI) database (http://ncbi.nlm.nih.gov/) or UCSC genome bioinformatics (http://genome.ucsc.edu/), and all the electropherograms were analyzed through visual inspection by an experienced researcher. The ‘already known’ *EGFR* mutations provided by the database were considered as mutation.

### MicroRNA extraction

MiRNA was extracted from the plasma of lung adenocarcinoma patients using the QiaGen miRNeasy Mini Kit (QiaGen, Hilden, Germany) according to the manufacturer’s instructions. Since no miRNA has been established as a ‘house-keeping gene’ in the plasma, we added 25 fmol of synthetic *C. elegans* miRNA *cel-miR-39* (Johnson & Johnson, Skillman, NJ, USA) in each plasma sample as an external control to monitor the quality of our RNA extraction and analysis.

### TaqMan low density array (TLDA)

TaqMan low density array (TLDA) was performed screening for miRNAs that differently profiled in different *EGFR* status. We choose 8 advanced stage (iii or iv) non-smoking female NCLC patients, including 4 *EGFR* mutation status positive and 4 negative which were paired with age. Each of these 8 samples was analyzed with an A & B card for duplicate detection of a total of 667 miRNAs, which including endogenous and negative controls. Each reaction included 50 ng of total RNA, 4.5 μl of reverse transcription (RT) reaction mixture (including 0.8 μl 10×Megaplex RT Primer Pools A+B 10×), 0.2 μl of dNTPs (100 nM), 1.5 μl of MultiScribe Reverse Transcriptase (50 U/μl), 0.8 μl of RT Buffer (10×), 0.9 μl of MgCl_2_ (25 mM), 0.1 μl of RNase inhibitor (20 U/μl) and 0.2 μl of nuclease-free water. Pre-amplification was performed after the RT procedure to increase the sensitivity of TLDA by using the TaqMan PreAmp Master mix and the Megaplex PreAmp Primer Pools A+B (Applied Biosystems, Foster City, CA). All the reactions were carried out according to the protocols recommended by the manufacturer on 7900HT theremocycler (Applied Biosystems, Foster City, CA). miRNAs that had RQ change larger than 1.5 or smaller than -1.5 in all of the four pairs were selected as candidate and further validated in a cohort.

### Reverse transcription

The extracted small RNA was reverse transcribed into its complementary DNA using the TaqMan microRNA Reverse Transcription Kit (Applied Biosystems, Foster City, CA). Each miRNA RT reaction included 1.5 μl of 10×RT buffer, 0.15 μl of 100 mM deoxynucleotide triphosphates, 1 μl of 50 U/μl MultiScribe Rever Transcriptase (Applied Biosystems, Foster City, CA), 0.19 μl of rebonuclease inhibitor, 3 μl of RT primer and 9.16 μl total RNA template. The reaction tube was incubated at 16°C for 30 minutes, 42°C for 30 minutes, 85°C for 5 minutes and finally 4°C for 5minutes. The RT reactions were multiple with 2 primers per RT.

### Quantitative real-time polymerase chain reaction (qRT-PCR)

QRT-PCR was carried out on the 7900HT thermocycler (Applied Biosystems, Foster City, CA). Each sample was tested in triplicate using the TaqMan microRNA assay (TaqMan Universal PCR Master Mix ii, no AmpErase UNG) (Applied Biosystems, Foster City, CA) (Assay IDs were shown in Table S2) along with the TaqMan probes and primers (Applied Biosystems, Foster City, CA). 15μl of PCR master mix of the PCR reactions started at 95°C for 10 minutes, followed by 50 cycles of 95°C for 15 seconds and 60°C for 60 seconds. The average quantification cycle (Cq) and standard deviation were calculated with the SDS software, along with the coefficient of variation (CV) of each sample. Samples with CV>0.05 were tested again. In our miRNA test all the samples had a CV<0.01. Samples with Cq<15 or Cq>35 were excluded. Real-time PCR data were analyzed using the SDS software v2.3 (settings: automatic baseline; threshold, 0.2) and relative miRNA levels were calculated with the RQ Manager v1.2.1 (Applied Biosystems, Foster City, CA). In addition, the raw Cq data obtained from each sample were normalized to the mean expression of spike-in *cel-miR-39* with the 2^-ΔΔCq^ transition.

### Statistical analysis

The associations of overall survival with miRNA expression and clinical factors were first estimated by the Kaplan-Meier survival analysis and log-rank test. The Cox proportional hazard regression models were then used to calculate the hazard ratios of miRNA and other risk factors. All the statistical analyses were performed using the SAS software version 9.2 (SAS Institute, Cary, NC, USA). *P* values less than 0.05 (two-sided) were considered statistically significant.

To assess the prediction accuracy of different miRNAs in the Cox regression model, time-dependent (ROC) curves for censored data and resulting area under the curve (AUC) were constructed according to Heagerty et al[11]. The Cox models with one, two or three selected miRNAs as covariates were fitted, and the risk scores were used to generate time-dependent sensitivity and specificity for the corresponding ROC curve at each observed event time. The AUC curve was plotted overtime to assess the prediction accuracy of the model in distinguishing subjects who have an event before detection from those who do not. Time-dependent ROC analyses were implemented using the R software (version 2.15.2) [12] and survival ROC package (version 1.0.3) [13] .

## Results

### Plasma miRNA expression and EGFR mutation

We profiled miRNAs extracted from the plasma of different *EGFR* mutation status lung adenocarcinoma patients (non-smoking female) using TLDA. Selected candidates identified in the initial screen were further validated in an extended study cohort of 105 non-smoking female lung adenocarcinomas. Table 1 showed the characteristics and clinical feature of the study cohort. Table 2 shows the expression of 20 miRNAs we tested in plasma. Since levels of expression varied widely among the miRNAs being analyzed, the raw Cq data for a specific miRNA were normalized to the mean raw Cq of this miRNA (or spike-in *cel-miR-39*) in all the 20 miRNA tested to get a ΔCq. Then ΔΔCq was defined as the difference between miRNA _specific ΔCq_ and *cel-miR-39*_ΔCq_. Expression levels were calculated using the 2^-ΔΔCq^ transition. However, none of the miRNA levels were significantly different between patients with and without mutant *EGFR* expect for *miR*-122(*P*=0.018). Furthermore, dividing the patients into early and late stage (stage I/II versus III/IV), we found the associations of *miR-16*, *miR-20b*, *miR-195*, *miR-122* and *miR-486-3p* with *EGFR* mutation status were evident in advanced stage (Table 3) (*P*=0.019, 0.047, 0.041, 0.033 or 0.017, respectively).

**Table 1:** Patient characteristics and clinical features.

**Variable**	**Number(%) n=105**	**HR (95%CI)**	**HR^*a*^(95%CI)**	**Log-rank *P***
Age				
<58	51(48.57%)	1.00	1.00	0.379
>=58	54(51.43%)	0.69(0.31-1.56)	0.73(0.32-1.66)	
Stage				
Early(Ⅰ/Ⅱ)	54(51.43%)	1.00	1.00	**0.010**
Late(Ⅲ/Ⅳ,)	51(48.57%)	**2.99(1.24-7.22)**	**3.13(1.23-7.98)**	
*EGFR*				
Wild	48(45.71%)	1.00	1.00	0.068
Mutant	57(54.29%)	2.22(0.92-5.36)	1.64(0.63-4.26)	
Family History**^*b*^**				
No	92(87.62%)	1.00	1.00	0.184
Yes	13(12.38%)	0.28(0.04-2.09)	0.25(0.03-1.87)	
Disease History**^*c*^**				
No	96(91.43%)	1.00	1.00	0.946
Yes	9(8.57%)	1.05(0.24-4.50)	0.93(0.21-4.04)	
Distant metastasis				
No	73(69.52%)	1.00	1.00	**1.000×10^-4^**
Yes	32(30.48%)	**4.48(1.91-10.49)**	**4.13(1.42-11.99)**	
Lymph Node metastasis				
No	52(49.52%)	1.00	1.00	**0.016**
Yes	53(50.48%)	**2.82(1.17-6.82)**	**2.02(0.71-5.72)**	
Treatment regimen				
Surgery	46(43.81%)	1.00	1.00	0.374
Surgery+Chemotherapy or Surgery+Radiation or Surgery+EGFR-TKIs	59(56.19%)	1.47(0.63-3.43)	3.82(0.30-49.00)	

a: adjusted for stage, age and treatment regimen

b: family history of cancer

c: lung disease history

**Table 2:** Plasma levels of miRNA expression by *EGFR* mutation status.

**MiRNA expression**	**Median (range)**	**(2^-ΔΔCq^)^*a*^**	***P* value**
	***EGFR* wild (n=48)**	***EGFR* mutant (n=57)**	
*miR-155*	0.91(0.28-6.67)	1.12(0.29-3.18)	0.557
*miR-25*	1.15(0.14-11.67)	0.89(0.12-9.82)	0.339
*miR-16*	1.17(0.13-8.56)	0.83(0.08-11.21)	0.087
*miR-133a*	0.88(0.07-9.64)	1.37(0.09-10.56)	0.077
*miR-19a*	1.21(0.22-6.05)	1.11(0.02-12.91)	0.221
*miR-19b*	1.16(0.23-6.14)	1.04(0.04-9.57)	0.310
*miR-20a*	1.06(0.27-8.46)	1.05(0.02-8.35)	0.332
*miR-20b*	1.09(0.16-12.06)	0.85(0.05-11.89)	0.165
*miR-629*	0.92(0.16-7.01)	0.91(0.16-9.18)	0.665
*miR-451*	1.08(0.08-13.95)	0.90(0.02-11.40)	0.114
*miR-192*	0.92(0.23-9.66)	0.91(0.14-9.57)	0.295
*miR-195*	1.13(0.22-8.98)	0.93(0.03-14.36)	0.133
***miR-122***	1.11(0.26-14.47)	0.84(0.04-21.14)	**0.018**
*miR-106b*	1.01(0.17-9.79)	1.05(0.11-6.07)	0.946
*miR-26b*	1.09(0.13-8.47)	1.10(0.04-5.46)	0.972
*miR-143*	1.05(0.20-17.34)	1.07(0.04-3.70)	0.615
*miR-374*	1.27(0.11-10.52)	1.11(0.02-7.93)	0.528
*miR-374-5p*	1.11(0.08-7.98)	0.99(0.01-7.93)	0.820
*miR-486-3p*	1.13(0.14-8.72)	0.75(0.06-9.97)	0.093
*miR-590-5p*	1.06(0.06-4.15)	1.04(0.09-8.52)	0.885

a: Expression level were calculated using the 2**^*-*^** ^ΔΔCq^ method.

miR-A_ΔCq_ = miR-A _raw Cq_- miR-A_mean Cq_,

miR-*A*_ΔΔCq_= *miR-A*_ΔCq_- *cel-miR-39*_ΔCq_

**Table 3:** Plasma levels of miRNA expression by *EGFR* mutation status (Stage iii/iv).

**miRNA expression**	**Median (range)**	**(2^-ΔΔCq^)^*a*^**	***P* value**
	***EGFR* wild (n=19)**	***EGFR* mutant (n=32)**	
*miR-155*	0.73(0.39-2.34)	1.03(0.29-3.02)	0.568
*miR-25*	0.88(0.26-11.67)	0.62(0.12-7.35)	0.078
***miR-16***	1.18(0.26-7.46)	0.75(0.08-7.08)	**0.019**
*miR-133a*	0.90(0.14-8.31)	1.16(0.09-10.56)	0.229
*miR-19a*	1.24(0.23-4.13)	0.99(0.02-6.61)	0.081
*miR-19b*	0.93(0.27-4.72)	0.92(0.04-5.99)	0.201
*miR-20a*	1.08(0.27-4.15)	0.87(0.02-8.35)	0.128
***miR-20b***	1.09(0.34-6.83)	0.71(0.05-9.09)	**0.047**
*miR-629*	0.93(0.31-7.01)	0.78(0.16-5.07)	0.170
*miR-451*	1.05(0.14-13.95)	0.63(0.02-11.40)	0.069
*miR-192*	0.96(0.26-9.66)	0.74(0.14-6.94)	0.091
***miR-195***	1.21(0.23-5.38)	0.62(0.03-8.81)	**0.041**
***miR-122***	1.03(0.32-9.77)	0.65(0.04-8.53)	**0.033**
*miR-106b*	0.92(0.17-7.79)	0.86(0.11-6.07)	0.992
*miR-26b*	1.01(0.13-5.16)	1.07(0.04-5.46)	0.961
*miR-143*	1.09(0.20-2.58)	1.03(0.04-3.51)	0.884
*miR-374*	1.29(0.11-2.93)	1.01(0.03-7.93)	0.749
*miR-374-5p*	1.09(0.08-3.37)	1.00(0.01-7.93)	0.839
***miR-486-3p***	1.08(0.32-8.72)	0.64(0.06-9.97)	**0.017**
*miR-590-5p*	1.07(0.28-3.91)	1.00(0.09-3.95)	0.445

a: Expression level were calculated using the 2**^*-*^** ^ΔΔCq^ method.

miR-A_ΔCq_ = miR-A _raw Cq_- miR-A_mean Cq_,

miR-*A*_ΔΔCq_= *miR-A*_ΔCq_- *cel-miR-39*_ΔCq_

### Patient characteristics and clinical features

This study was based on 105 non-smoking female lung adenocarcinoma patients with a median follow-up of 23 months (range 4-58 months). Of all the patients enrolled, there were 24 deaths and 6 losses to follow-up. The mean age of patients at diagnosis was 58.60±9.65 years and the range was between 34 and 80 years. The distributions of patient characteristics and clinical features were summarized in Table 1. Log-rank test suggested that patients with advanced disease stage(stage iii/iv), distant metastasis, lymph node involvement lung adenocarcinoma had a significantly shorter overall survival compared to those without these features (*P*=0.010, 1.0×10^-4^, or 0.016, respectively). Cox regression analysis showed that the risk for death was associated with distant metastasis (HR=4.13, 95%CI=1.42-11.99) or lymph node metastasis (HR=2.02, 95%CI=0.71-5.72) after adjusting for age, stage, treatment regimen. However, no differences in survival time were observed between patients with different age, *EGFR* mutation status, a family history of cancer, a history of lung diseases and treatment regimen.

### Plasma miRNA expression and non-smoking female lung adenocarcinoma survival

The relationships between 20 miRNA expression in plasma and the survival of non-smoking female lung adenocarcinoma patients are shown in Table 4. We grouped the patients into two classes (low versus high expression) based on the median level of expression in each miRNA. Our survival analysis found that among the 20 miRNAs *miR-19a*, *miR-19*5 and *miR-122* were significantly associated with overall survival (*P*=0.040, 0.011 and 0.012, respectively). High expression of *miR-19a*, *miR-19*5 and *miR-122* in plasma were associated with lower risks of death compared to low expression. The hazards ratios (HRs) were 0.41, 0.30 and 0.33, respectively. These associations, except for *miR-19a*, remained significant after adjusting for stage, age, treatment regimen (including surgey, surgey+chemotherapy, surgey+radiation, surgey+EGFR-TKIs).

**Table 4:** miRNA expression and survival in non-smoking female lung adenocarcinoma.

**miRNAs**	**group**	**Number n =105**	**HR (95% CI)**	**HR ^*a*^(95% CI)**	**Log-rank *P***
*miR-155*	LE	52	1.00	1.00	0.533
	HE	53	1.29(0.58-2.89)	1.38(0.61-3.13)	
*miR-25*	LE	53	1.00	1.00	0.160
	HE	52	0.55(0.23-1.29)	0.68(0.28-1.62)	
*miR-16*	LE	52	1.00	1.00	0.184
	HE	53	0.57(0.24-1.33)	0.61(0.26-1.47)	
*miR-133a*	LE	52	1.00	1.00	0.280
	HE	53	1.57(0.69-3.62)	1.86(0.79-4.35)	
***miR-19a***	LE	52	1.00	1.00	**0.040**
	HE	53	**0.41(0.17-0.99)**	0.41(0.17-1.00)	
*miR-19b*	LE	52	1.00	1.00	0.095
	HE	53	0.49(0.21-1.16)	0.51(0.22-1.22)	
*miR-20a*	LE	53	1.00	1.00	0.218
	HE	52	0.59(0.25-1.39)	0.68(0.28-1.63)	
*miR-20b*	LE	52	1.00	1.00	0.180
	HE	53	0.56(0.24-1.32)	0.63(0.26-1.51)	
*miR-629*	LE	52	1.00	1.00	0.218
	HE	53	0.60(0.26-1.37)	0.61(0.27-1.39)	
*miR-451*	LE	52	1.00	1.00	0.335
	HE	53	0.67(0.29-1.53)	0.79(0.34-1.86)	
*miR-192*	LE	51	1.00	1.00	0.093
	HE	54	0.49(0.21-1.15)	0.55(0.23-1.31)	
***miR-195***	LE	53	1.00	1.00	**0.011**
	HE	52	**0.30(0.11-0.80)**	**0.31(0.11-0.83)**	
***miR-122***	LE	53	1.00	1.00	**0.012**
	HE	52	**0.33(0.13-0.82)**	**0.34(0.13-0.88)**	
*miR-106b*	LE	53	1.00	1.00	0.090
	HE	52	0.49(0.21-1.14)	0.49(0.20-1.21)	
*miR-26b*	LE	52	1.00	1.00	0.831
	HE	53	0.92(0.41-2.06)	0.98(0.41-2.31)	
*miR-143*	LE	53	1.00	1.00	0.497
	HE	52	1.32(0.59-2.99)	1.29(0.54-3.06)	
*miR-374*	LE	52	1.00	1.00	0.892
	HE	53	1.06(0.47-2.39)	1.03(0.44-2.43)	
*miR-374-5p*	LE	52	1.00	1.00	0.887
		HE	53	0.94(0.42-2.12)	0.92(0.39-2.18)
*miR-486-3p*	LE	53	1.00	1.00	0.096
	HE	52	0.48(0.20-1.17)	0.55(0.23-1.36)	
*miR-590-5p*	LE	52	1.00	1.00	0.052
	HE	53	0.44(0.19-1.03)	0.43(0.18-1.02)	

LE: Low Expression; HE: High Expression. LE and HE were divided by median expression of specific miRNA.

a adjusted for stage, age and treatment

Furthermore, dividing the patients into early and late stage (stage I/II versus III/IV), we found the associations of *miR-19a*, *miR-195* and *miR-122* with survival were evident in advanced stage (Table 5). Among the patients with stage iii or iv diseases, high expression of *miR-19a*, *miR-195* and *miR-122* were significantly associated with a low risk of death (adjusted HR=0.26, 95%CI: 0.08-0.83; adjusted HR=0.23, 95%CI: 0.07-0.84, and adjusted HR=0.22, 95%CI: 0.06-0.77, respectively). Interestingly, we also found significant relationships between miRNA expression and survival among patients with mutant *EGFR* (Table 6). Since patients with *EGFR* mutation were likely to receive target therapy, which could be a confounding factor, we performed the Cox regression analysis adjusted for the treatment regimen in addition to stage and age. Our results indicated that high expression of *miR-19*a, *miR-19b*, *miR-195*, *miR-122* and *miR-590-5p* in plasma were associated with better overall survival among non-smoking female lung adenocarcinoma patients who had *EGFR* mutation.

**Table 5:** miRNA expression and survival in non-smoking female lung adenocarcinoma by stage.

**Stage**	**miRNAs**	**group**	**Number**	**HR (95% CI)**	**HR^*a*^(95% CI)**	**Log-rank *P***
Ⅰ/Ⅱ	*miR-19a*	LE	27	1.00	1.00	0.543
		HE	27	0.58(0.13-2.59)	0.51(0.11-2.37)	
	*miR-195*	LE	26	1.00	1.00	0.229
		HE	28	0.38(0.07-1.96)	0.40(0.08-2.04)	
	*miR-122*	LE	24	1.00	1.00	0.599
		HE	30	0.67(0.15-3.01)	0.82(0.17-3.93)	
Ⅲ/Ⅳ	*miR-19a*	LE	25	1.00	1.00	**0.047**
		HE	26	0.34(0.11-1.04)	**0.26(0.08-0.83)**	
	*miR-195*	LE	27	1.00	1.00	**0.037**
		HE	24	0.29(0.08-1.00)	**0.23(0.07-0.84)**	
	*miR-122*	LE	29	1.00	1.00	**0.019**
		HE	22	**0.25(0.07-0.88)**	**0.22(0.06-0.77)**	

LE: Low Expression; HE: High Expression. LE and HE were divided by median expression of specific miRNA.

a adjusted for stage, age and treatment

**Table 6:** miRNA expression and survival in non-smoking female lung adenocarcinoma by *EGFR* mutation status.

***EGFR***	**miRNAs**	**group**	**Number**	**HR (95% CI)**	**HR^*a*^(95% CI)**	**Log-rank *P***
Wild	*miR-19a*	LE	23	1.00	1.00	0.686
		HE	25	0.74(0.16-3.30)	0.73(0.16-3.34)	
	*miR-19b*	LE	22	1.00	1.00	0.857
		HE	26	1.15(0.26-5.13)	1.20(0.27-5.41)	
	*miR-195*	LE	22	1.00	1.00	0.261
		HE	26	0.40(0.08-2.09)	0.41(0.08-2.19)	
	*miR-122*	LE	21	1.00	1.00	0.671
		HE	27	0.73(0.16-3.26)	0.84(0.16-4.37)	
	*miR-590-5p*	LE	23	1.00	1.00	0.673
		HE	25	0.73(0.16-3.25)	0.68(0.15-3.11)	
mutant	*miR-19a*	LE	29	1.00	1.00	**0.026**
		HE	28	0.30(0.10-0.92)	0.28(0.09-0.88)	
	*miR-19b*	LE	30	1.00	1.00	**0.033**
		HE	27	0.32(0.10-0.97)	0.28(0.09-0.91)	
	*miR-195*	LE	31	1.00	1.00	**0.034**
		HE	26	0.28(0.08-0.99)	0.27(0.08-0.96)	
	*miR-122*	LE	32	1.00	1.00	**0.008**
		HE	25	0.22(0.06-0.75)	0.23(0.06-0.81)	
	*miR-590-5p*	LE	29	1.00	1.00	**0.021**
		HE	28	0.31(0.11-0.89)	0.28(0.09-0.85)	

LE: Low Expression; HE: High Expression. LE and HE were divided by median expression of specific miRNA.

a adjusted for stage, age and treatment

**Figure 1:**
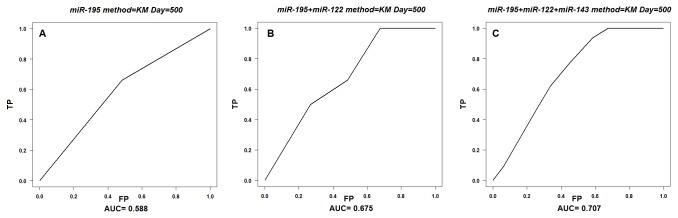
ROC curves for the model score, AUC based on Cox model. A: one covariate model ROC curves based on the risk score of *miR-195*; B: two covariate model ROC curves based on the risk score of *miR-195* and miR-122; C: three covariate model ROC curves based on the risk score of *miR-195*, miR-122 and *miR-143*.

**Figure 2:**
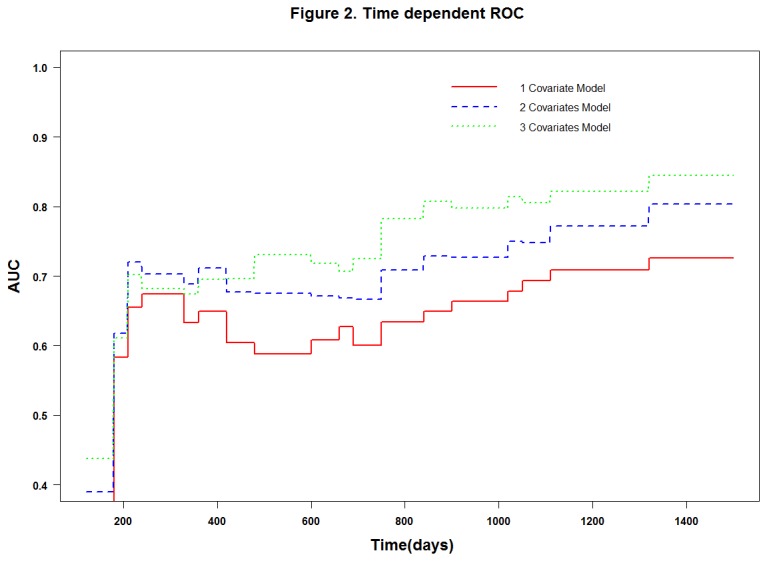
Time-dependent ROC curves of three covariate models. AUC(t) based on one, two and three covariate model overtime under Cox model, when after 500 days three covariate Cox model was the most predictive one, which achieved more than 70% of AUC.

By using Cox proportional hazards regression models we got three model to help calculate the risk score of patient survival. One covariate model was conducted by *miR-195* expression (Risk Score=-1.21×*miR-195exp*), while two covariates model including *miR-195* and *miR-122* expression (Risk Score=-1.04×*miR-195exp*-0.96×*miR-122exp*), in three covariates model besides *miR-195* and *miR-122*, *miR-143* was also included (Risk Score = -1.26×*miR-195exp*-1.34×*miR-122exp*+1.09×*miR-143exp*). In order to evaluate the accuracy of survival prediction in the 105 non-smoking female lung cancer patients, we plot the time-dependent ROC curve. The AUC (500 days) was 0.588 for the one-covariate Cox regression model (Figure 1A), 0.675 for a two-covariate Cox model (Figure 1B) and 0.707 for a three-covariate model (Figure 1C). In addition, since the accuracy of each model may change over time, we plotted the AUC curves overtime under each Cox model (Figure 2). In general, we found that the three-covariate Cox model was the most predictive one for non-smoking female lung adenocarcinoma patients as it achieved more than 70% of AUC after 500 days.

## Discussion

According to the estimation by WHO, 25% of lung cancer occurs to never smokers worldwide [14]. Lung cancer is the top 10 cause of death among non-smokers in the world, and the 9th leading cause of cancer death in women according to the data from the European cancer registries[1]. Non-smoking female lung cancer is considered a unique disease entity. The genetic and environmental attributes to this malignancy, however, remain unclear [15]. Our study showed that advanced disease stage and tumor metastasis(distant metastasis and lymph node metastasis) were independent risk factors for the poor prognosis of non-smoking female lung adenocarcinoma patients. These findings were consistent with the literature[16]. Furthermore, our additional analyses indicated that plasma levels of *miR-122* expression were associated with *EG*FR mutation status along with the overall survival of these patients. Especially in those with advanced disease stage, *miR-195* and *miR-122* expression were found to be associated with *EGFR* mutation and overall survival of lung adenocarcinoma. Moreover, for the patients with *EGFR* mutation, *miR-19a, miR-19b* and *miR-590-5p* expression were also found to be associated with overall survival in addition to *miR-195* and *miR-122*.

Disease stage at diagnosis is known to be a major prognostic determinant[16], but differences in survival still exist among patients with similar disease stage, which is explained by marked heterogeneity in the genetic and molecular background of the disease[17]. The pathogenesis of lung cancer differs substantially from patient to patient, including their smoking status. Distinct genomic profiles regarding oncogene mutation (*EGFR*) and patient response to targeted therapy indicate the possible cellular and molecular differences. The possible effect of sex hormones on lung cancer is considered another distinct molecular aspect because adenocarcinoma is diagnosed more frequently in Asian women who do not smoke than in men. A recent study also suggests that different molecular profiles of estrogen receptor subtypes α and β may affect the survival outcomes of lung cancer [18]. All these indicate sexual hormones may influence the disease process.

Advance in genomics, epigenomics and molecular pathology has led to the identification of new biomarkers for cancer detection and outcome prediction. MicroRNA is an expansion to the existing pool of oncogenes and tumor suppressor genes. Emerging evidence indicates that alterations of miRNA expression may affect the initiation and progression of cancer. A number of miRNAs have been reported to be associated with clinical outcomes of patients with cancer, including chronic lymphocytic leukemia[19], breast cancer [20], and lung cancer[21,22]. Although our knowledge on the role of miRNA expression in cancer is still limited, evidence suggests that miRNAs may serve as a new class of clinical biomarker for cancer prognosis. For example, reduced expression of let-*7* in lung cancer has been found to be associated with poor survival [22]. The prognostic values of let-*7*, along with *miR-155* and let-*7a*-*2* in lung cancer were also demonstrated in another study [23].

A large number of miRNAs have been found to be stably expressed in human serum and plasma. Circulating miRNAs and their expression profiles have been proposed to be useful biomarkers in the diagnosis and prognosis of cancer. Hu et al.first found that the expression of four serum miRNAs(*miR-486*, *miR-30d*, *miR-1* and *miR-499*) were significantly associated with overall survival of NSCLC patients[24]. Over-expression of serum *miR-21* was linked to the poor survival of NSCLC and associated with lymph node metastasis and advanced stage [25]. In addition, several other miRNAs have been reported to be associated with clinical outcomes of NSCLC patients, including *miR-30e-3p*, let-*7f*[26], *miR-126*, *miR-183*[27], *miR-125b*[28], and *miR-17-5p*[29]. Recently, Wang et al. reported a group of *TGF-β* signaling pathway-related serum miRNA as predictors of survival in advanced NSCLC, which including *miR-16* [30]. 

Few studies have investigated the prognosis of NSCLC in non-smoking female patients. In order to improve the management of these patients, we focus our study on non-smoking female lung adenocarcinoma in searching for molecular markers that can identify patients who have poor prognosis after surgery. Circulating miRNAs are good candidates for non-invasive blood-based test in prediction of lung cancer prognosis. The results of our study provided some evidence that plasma levels of *miR-195* and *miR-122* expression were associated with *EGFR* status along with overall survival of non-smoking female lung adenocarcinoma patients, especially in those with advanced stage or *EGFR* mutation positive. *MiR-195* is located on chromosome 17p13.1, and belongs to the *miR-16/15/195/424/497* family which shares the same 3’UTR binding seed sequence. A number of studies have shown that this family plays an important role in tumorigenesis. *MiR-195* has been found in several types of cancer, and recently the ectopic expression of *miR-195* was found in hepatocellular carcinoma and colorectal cancer cells [31,32]. Xu et al. reported that *miR-195* could block the G(1)/S transition by interfering with the *Rb-E2F* signaling pathway through targeting multiple molecules (*cyclin D1*, *CDK6*, and *E2F3*)[31]. Furthermore, over-expression of *miR-195* in the *Rb-E2F* pathway which acts as a vital check point in cell cycle progression, can promote cell proliferation, which may subsequently facilitate the development of cancer. Similarly, Liu et al. revealed that the *miR-15/16/195* directly targeted *cyclin D1* and *CDK6*, and *miR-195* expression increased the proportion of G1 cells in a lung cancer cell line[32]. In addition, circulating levels of *miR-195* may be a biomarker for minimal invasion of breast cancer[33]. Our results indicated that reduced expression of *miR-195* in plasma could lead to better survival of non-smoking female lung adenocarcinoma patients, especially for those with advanced stage. Despite the consistent findings, the mechanism that underlies the effect of *miR-195* on lung cancer is still unclear. Our study was one of the few investigations which focused on non-smoking female lung adenocarcinoma. Our finding of association between circulating *miR-195* and non-smoking female lung adenocarcinoma survival warrants further elucidation.

*MiR-122*, located on chromosome 18q21.3, is a liver-specific miRNA representing two thirds of hepatic miRNAs. This miRNA was reported to facilitate the replication of hepatitis C virus[34] and regulate the metabolism of lipids[35] and expression of hepatic circadian genes[36]. Notably, down-regulation of *miR-122* has been associated with human hepatocellular carcinoma development and progression [37]. We investigated the relationship between *miR-122* and *EGFR* mutation status along with survival in non-smoking female lung adenocarcinoma, and found that reduced expression of *miR-122* in plasma was associated with *EGFR* mutation and favorable survival, especially in patients with advanced disease. To date, neither the mechanisms underlying *miR-122* regulation nor the regulatory networks of *miR-122* have been investigated. Recently, a few targets of *miR-122* have been investigated including *cyclin G1*, serum response factor, insulin-like growth factor 1 receptor (*IGF-1R*)[37–39]. *MiR-122* may have influence on survival of lung adenocarcinoma by targeting these gene or through the *GSK-3b–C/EBPa–miR-122–IGF-1R* regulatory circuitry[40].

Interestingly, we identified a group of miRNAs (including *miR-19a*, *miR-19b*, *miR-195*, *miR-122* and *miR-590-5p*) that could be predictive of survival outcomes of non-smoking female lung adenocarcinoma patients who have *EGFR* mutations in Exons 18-21. It has been suggested that the carcinogenic pathway of lung cancer may differ by smoking status. The differences between smoking and non-smoking lung adenocarcinoma are found at cellular and molecular levels [41], including distinct profiles of oncogenic mutations (e.g., *EGFR*). Our results suggest that *miR-19a*, *miR-19b*, *miR-195*, *miR-122* and *miR-590-5p* may predict the prognosis of lung adenocarcinoma with mutant type of *EGFR* in non-smoking females. Furthermore, these results provide new evidence that *EGFR* mutant lung adenocarcinoma may have distinct miRNAs associated with overall survival. In this group of miRNAs, *miR-195* and *miR-122* was also differently expressed in *EGFR* wild and *EGFR* mutant groups. Which indicated that *miR-195* and *miR-122* may possibly be potential non-invasive biomarker in *EGFR* mutation prediction and prognosis prediction of non-smoking female lung adenocarcinomas. These findings need to be confirmed in large well-designed clinical studies.

To our knowledge, this study represents the first effort to characterize the relationships of plasma miRNAs with *EGFR* mutation and lung adenocarcinoma survival in non-smoking women. We measured plasma levels of 20 miRNAs and analyzed their associations with lung adenocarcinoma survival. Our study also evaluated the association in patients stratified by clinical stage and status of *EGFR* mutation, which was valuable for assessing possible interaction. We also used different analytic strategies like survival ROC analysis to confirm our results. However, our study also had a few limitations. First, although we performed subgroup analysis according to clinical stage and *EGFR* status and calculation of adjusted HRs, confounding factors may still exist in our study. Second, we found associations between overall survival and plasma miRNA expression with little knowledge on their biologic functions. Functional analyses of these miRNAs are needed to establish their biological relevance. Third, this is a relatively small study. Our findings need to be validated in large well-powered studies independently.

In conclusion, our study showed that circulating miRNAs were associated with *EGFR* status and overall survival of lung adenocarcinoma. High expression of *miR-195* and *miR-122* in plasma could help predict *EGFR* mutation status and overall survival of advanced stage female non-smokers with lung adenocarcinoma. Furthermore, the association between plasma miRNAs and overall survival of lung adenocarcinoma may differ by the status of *EGFR* mutation.

## Supporting Information

Table S1 **EGFR Exon18-21 PCR Primers.** Table S1shown the *EGFR* primers. DNA for *EGFR* sequencing test was extracted from the surgical specimen using the pheno-chloroform method. PCR was performed on 100 ng DNA samples to identify mutations in the *EGFR* exons 18-21.
(DOCX)

Table S2 **Assay IDs for the microRNA assays (Applied Biosystems, Foster City, CA).** Each sample was tested in triplicate using the TaqMan microRNA assays, the assay IDs were shown in Table S2 along with the TaqMan probes and primers (Applied Biosystems, Foster City, CA).
(DOCX)
