# Supplementary material for: Circulating MicroRNAs in Relation to EGFR Status and Survival of Lung Adenocarcinoma in Female Non-Smokers
Source: PLoS One. 2013 Nov 25;8(11):e81408. doi: 10.1371/journal.pone.0081408 (PMC3839880; doi:10.1371/journal.pone.0081408)
Supplement: Table S1 — EGFR Exon18-21 PCR Primers. Table S1shown the EGFR primers. DNA for EGFR sequencing test was extracted from the surgical specimen using the pheno-chloroform method. PCR was performed on 100 ng DNA samples to identify mutations in the EGFR exons 18-21. (DOCX) [file pone.0081408.s001.docx]

**Table S1. *EGFR* Exon18-21 PCR Primers**

| **Exon** | **Primers** |
| --- | --- |
| 18 | 5’-ATGGTGAGGGCTGAGGTGAC -3’ |
|  | 5’-GGGCTCCACGAATCACACTG -3’ |
| 19 | 5’-CGTCACAGCCCCCAGCAA -3’ |
|  | 5’-CCCCACACAGCAAAGCAGAA -3’ |
| 20 | 5’- GTCACTTCACAGCCCTGCGT -3’ |
|  | 5’- CTCCCCGTATCTCCCTTCCC -3’ |
| 21 | 5’- ATTCGGATGCAGAGCTTCTT -3’ |
|  | 5’- TGGTCCCTGGTGTCAGGAAA -3’ |

**Table S2. Assay IDs for the microRNA assays (Applied Biosystems, Foster City, CA)**

| **Assay ID** | **Assay Name** | **Target Sequence** |
| --- | --- | --- |
| 002623 | *hsa-miR-155* | UUAAUGCUAAUCGUGAUAGGGGU |
| 000403 | *hsa-miR-25* | CAUUGCACUUGUCUCGGUCUGA |
| 000391 | *hsa-miR-16* | UAGCAGCACGUAAAUAUUGGCG |
| 002246 | *hsa-miR-133a* | UUUGGUCCCCUUCAACCAGCUG |
| 002245 | *hsa-miR-122* | UGGAGUGUGACAAUGGUGUUUG |
| 000395 | *hsa-miR-19a* | UGUGCAAAUCUAUGCAAAACUGA |
| 000396 | *hsa-miR-19b* | UGUGCAAAUCCAUGCAAAACUGA |
| 000580 | *hsa-miR-20a* | UAAAGUGCUUAUAGUGCAGGUAG |
| 001014 | *hsa-miR-20b* | CAAAGUGCUCAUAGUGCAGGUAG |
| 000407 | *hsa-miR-26b* | UUCAAGUAAUUCAGGAUAGGU |
| 000442 | *hsa-miR-106b* | UAAAGUGCUGACAGUGCAGAU |
| 002249 | *hsa-miR-143* | UGAGAUGAAGCACUGUAGCUC |
| 001141 | *hsa-miR-451* | AAACCGUUACCAUUACUGAGUU |
| 002436 | *hsa-miR-629* | UGGGUUUACGUUGGGAGAACU |
| 000491 | *hsa-miR-192* | CUGACCUAUGAAUUGACAGCC |
| 000494 | *hsa-miR-195* | UAGCAGCACAGAAAUAUUGGC |
| 000563 | *hsa-miR-374a* | UUAUAAUACAACCUGAUAAGUG |
| 001319 | *hsa-miR-374b* | AUAUAAUACAACCUGCUAAGUG |
| 002093 | *hsa-miR-486-3p* | CGGGGCAGCUCAGUACAGGAU |
| 001984 | *hsa-miR-590-5p* | GAGCUUAUUCAUAAAAGUGCAG |
