# Supplementary material for: Circulating MicroRNAs in Relation to EGFR Status and Survival of Lung Adenocarcinoma in Female Non-Smokers
Source: PLoS One. 2013 Nov 25;8(11):e81408. doi: 10.1371/journal.pone.0081408 (PMC3839880; doi:10.1371/journal.pone.0081408)
Supplement: Table S2 — Assay IDs for the microRNA assays (Applied Biosystems, Foster City, CA). Each sample was tested in triplicate using the TaqMan microRNA assays, the assay IDs were shown in Table S2 along with the TaqMan probes and primers (Applied Biosystems, Foster City, CA). (DOCX) [file pone.0081408.s002.docx]

**Table S2. Assay IDs for the microRNA assays (Applied Biosystems, Foster City, CA)**

| **Assay ID** | **Assay Name** | **Target Sequence** |
| --- | --- | --- |
| 002623 | *hsa-miR-155* | UUAAUGCUAAUCGUGAUAGGGGU |
| 000403 | *hsa-miR-25* | CAUUGCACUUGUCUCGGUCUGA |
| 000391 | *hsa-miR-16* | UAGCAGCACGUAAAUAUUGGCG |
| 002246 | *hsa-miR-133a* | UUUGGUCCCCUUCAACCAGCUG |
| 002245 | *hsa-miR-122* | UGGAGUGUGACAAUGGUGUUUG |
| 000395 | *hsa-miR-19a* | UGUGCAAAUCUAUGCAAAACUGA |
| 000396 | *hsa-miR-19b* | UGUGCAAAUCCAUGCAAAACUGA |
| 000580 | *hsa-miR-20a* | UAAAGUGCUUAUAGUGCAGGUAG |
| 001014 | *hsa-miR-20b* | CAAAGUGCUCAUAGUGCAGGUAG |
| 000407 | *hsa-miR-26b* | UUCAAGUAAUUCAGGAUAGGU |
| 000442 | *hsa-miR-106b* | UAAAGUGCUGACAGUGCAGAU |
| 002249 | *hsa-miR-143* | UGAGAUGAAGCACUGUAGCUC |
| 001141 | *hsa-miR-451* | AAACCGUUACCAUUACUGAGUU |
| 002436 | *hsa-miR-629* | UGGGUUUACGUUGGGAGAACU |
| 000491 | *hsa-miR-192* | CUGACCUAUGAAUUGACAGCC |
| 000494 | *hsa-miR-195* | UAGCAGCACAGAAAUAUUGGC |
| 000563 | *hsa-miR-374a* | UUAUAAUACAACCUGAUAAGUG |
| 001319 | *hsa-miR-374b* | AUAUAAUACAACCUGCUAAGUG |
| 002093 | *hsa-miR-486-3p* | CGGGGCAGCUCAGUACAGGAU |
| 001984 | *hsa-miR-590-5p* | GAGCUUAUUCAUAAAAGUGCAG |
